# Unveiling the Efficacy, Safety, and Tolerability of Anti-Interleukin-1 Treatment in Monogenic and Multifactorial Autoinflammatory Diseases

**DOI:** 10.3390/ijms20081898

**Published:** 2019-04-17

**Authors:** Alessandra Bettiol, Giuseppe Lopalco, Giacomo Emmi, Luca Cantarini, Maria Letizia Urban, Antonio Vitale, Nunzio Denora, Antonio Lopalco, Annalisa Cutrignelli, Angela Lopedota, Vincenzo Venerito, Marco Fornaro, Alfredo Vannacci, Donato Rigante, Rolando Cimaz, Florenzo Iannone

**Affiliations:** 1 Department of Neurosciences, Psychology, Pharmacology and Child Health (NEUROFARBA), University of Florence, 50139 Florence, Italy; alessandra.bettiol@unifi.it (A.B.); alfredo.vannacci@unifi.it (A.V.); 2 Department of Experimental and Clinical Medicine, University of Florence, 50134 Florence, Italy; giacomaci@yahoo.it (G.E.); marialetiziaurban@hotmail.it (M.L.U.); 3 Department of Emergency and Organ Transplantation, Rheumatology Unit, University of Bari, 70121 Bari, Italy; vincenzo.venerito@gmail.com (V.V.); marco3987@hotmail.it (M.F.); florenzo.iannone@uniba.it (F.I.); 4 Department of Medical Sciences, Surgery and Neurosciences, University of Siena, 53100 Siena, Italy; cantariniluca@hotmail.com (L.C.); espositogenn@gmail.com (A.V.); 5 Department of Pharmacy-Drug Sciences, University of Bari “A. Moro”, 70125 Bari, Italy; nunzio.denora@uniba.it (N.D.); antonio.lopalco@uniba.it (A.L.); annalisa.cutrignelli@uniba.it (A.C.); angelaassunta.lopedota@uniba.it (A.L.); 6 Institute of Pediatrics, Fondazione Policlinico Universitario “A. Gemelli” IRCCS, 00168 Rome, Italy; drigante@gmail.com; 7 Institute of Pediatrics, Università Cattolica Sacro Cuore, 00198 Rome, Italy; 8 Department of Neurosciences, Psychology, Drug Research and Child Health, Rheumatology Unit, Meyer Children’s Hospital, University of Florence, 50139 Florence, Italy; r.cimaz@gmail.com

**Keywords:** Interleukin-1, anakinra, canakinumab, innovative biotechnologies, autoinflammatory disease, Kawasaki disease, systemic juvenile idiopathic arthritis, personalized medicine, child, pediatrics

## Abstract

Autoinflammatory diseases (AIDs) are heterogeneous disorders characterized by dysregulation in the inflammasome, a large intracellular multiprotein platform, leading to overproduction of interleukin-1(IL-1)β that plays a predominant pathogenic role in such diseases. Appropriate treatment is crucial, also considering that AIDs may persist into adulthood with negative consequences on patients’ quality of life. IL-1β blockade results in a sustained reduction of disease severity in most AIDs. A growing experience with the human IL-1 receptor antagonist, Anakinra (ANA), and the monoclonal anti IL-1β antibody, Canakinumab (CANA), has also been engendered, highlighting their efficacy upon protean clinical manifestations of AIDs. Safety and tolerability have been confirmed by several clinical trials and observational studies on both large and small cohorts of AID patients. The same treatment has been proposed in refractory Kawasaki disease, an acute inflammatory vasculitis occurring in children before 5 years, which has been postulated to be autoinflammatory for its phenotypical and immunological similarity with systemic juvenile idiopathic arthritis. Nevertheless, minor concerns about IL-1 antagonists have been raised regarding their employment in children, and the development of novel pharmacological formulations is aimed at minimizing side effects that may affect adherence to treatment. The present review summarizes current findings on the efficacy, safety, and tolerability of ANA and CANA for treatment of AIDs and Kawasaki vasculitis with a specific focus on the pediatric setting.

## 1. Introduction

Autoinflammatory diseases (AIDs) are a heterogeneous group of monogenic and multifactorial diseases characterized by recurrent or chronic inflammation caused by dysregulation of the innate immune system [1,2]. Most of these disorders have an early onset, ranging from the first hours to the first decade of life. However, due to their rarity, a diagnostic delay is frequently observed [3]. The main subgroup of AIDs includes different hereditary periodic fever syndromes, such as cryopyrin-associated periodic syndrome (CAPS), tumor necrosis factor receptor-associated periodic fever syndrome (TRAPS), hyperimmunoglobulin D syndrome/mevalonate kinase deficiency (HIDS/MKD), and familial Mediterranean fever (FMF) [2]. These conditions follow an autosomal dominant (CAPS and TRAPS) or recessive (HIDS/MKD and FMF) hereditary pattern and share a common clinical background marked by recurrent fever attacks and inflammation involving different sites, such as skin, serosal membranes, joints, gastrointestinal tract, or central nervous system [4,5]. AA amyloidosis is their most serious complication in the long-term, with an overall prevalence ranging from 2% to 25% of cases [6]. Many multifactorial disorders manifest with the same clinical features observed in inherited AIDs and even share the same pathogenic pathway [7]. In this regard, recurrent fevers with arthritis and cutaneous rashes are also typical features of systemic juvenile idiopathic arthritis (SJIA), which has been classified up to now as a category of juvenile idiopathic arthritis, the most common rheumatic disease in childhood. SJIA can lead to growth retardation, osteoporosis, and life-threatening complications, such as macrophage activation syndrome (MAS), and is now considered an autoinflammatory condition [8,9]. Recently, an autoinflammatory origin has also been suggested for Kawasaki disease (KD), an acute self-limited systemic vasculitis usually occurring in children before 5 years and involving medium-sized vessels, especially coronary arteries, which represents the first cause of childhood-acquired heart disease in developed countries [10]. Irrespective of the specific underlying pathways, these syndromes are characterized by an overproduction of interleukin (IL)-1, which initiates the inflammatory cascade and leads to tissue damage of variable degrees. Therefore, appropriate and effective treatment is crucial, considering that these conditions may persist into adulthood with negative consequences on both the patient’s future health and quality of life [11]. Monotherapy blocking IL-1 activity results in a sustained reduction of disease severity, but chronic treatment is often required to control inflammatory flares for the lifetime and prevent long-term complications. Therefore, monitoring the safety profile and tolerability of therapy in children affected by these disorders is of paramount importance since it may affect adherence to treatment and overall clinical efficacy. In this work, we aimed at providing current findings on the efficacy, safety, and tolerability of Anakinra (ANA) and Canakinumab (CANA) for treatment of AIDs and Kawasaki vasculitis with a specific focus on the pediatric setting.

## 2. IL-1 Blockade in Autoinflammatory Diseases

The IL-1 family includes key cytokines involved in the innate immune response as well as in the mechanisms of fever and inflammation [12,13]. Namely, IL-1 induces the synthesis of major inflammatory mediators, such as cyclooxygenase type 2 (COX-2), type 2 phospholipase A, and inducible nitric oxide synthase, which in turn account for the production of prostaglandin-E2, platelet activating factor, and nitric oxide [12]. The two major cytokines, IL-1α and IL-1β, exert their pro-inflammatory effects by binding the IL-1 family receptors, which are characterized by the presence of the Toll-IL-1 receptor (TIR) domain in the cytoplasmic portion [14,15]. In physiological conditions, both IL-1α and IL-1β bind to type 1 IL-1 receptor (IL-1R1) and to the adaptor protein, IL-1RAcP, in order to trigger signal transduction [16]. On the contrary, the IL-1 receptor antagonist (IL-1RA) is a competitive inhibitor that prevents IL-1α and IL-1β from interacting with the IL-1 receptor 1 (IL-1R1). Much less is instead known about the type 2 IL-1 receptor (IL-1R2), a decoy receptor for IL-1β, lacking a cytoplasmic domain, which does not have a signal role, but rather sequesters IL-1β [17]. Most AIDs are caused by a lacking regulation in the inflammasome, a large intracellular multiprotein platform, leading to an overproduction of IL-1β that plays a predominant pathogenic role in such disorders [18].

**Figure 1:**
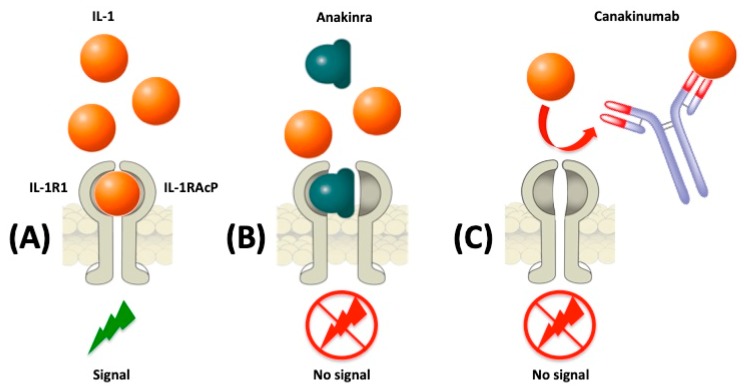
Strategies for IL-1 blockade with Anakinra and Canakinumab. (**A**) Interleukin (IL)-1 binds to type 1 IL-1 receptor (IL-1R1) and to the adaptor protein, IL-1RAcP, in order to trigger signal transduction. (**B**) The recombinant human IL-1R1 antagonist, Anakinra, directly competes with IL-1 for binding to the IL-1R1, blocking the biological activity of IL-1. (**C**) The human monoclonal IgG1 antibody, Canakinumab, selectively neutralizes IL-1β and inhibits its binding to the IL-1 receptor.

Four biologic drugs blocking IL-1 are currently available [19,20]. Of them, ANA and CANA have been approved for clinical use in Europe, whereas the soluble decoy IL-1-receptor, rilonacept, and the human-engineered monoclonal anti-IL-1β, gevokizumab, are not authorized in European countries. ANA is a human IL-1 receptor antagonist that acts by competitively inhibiting the binding of IL-1 with the IL-1 type 1 receptor [21]. ANA (Kineret^®^) is currently approved for the treatment of rheumatoid arthritis (RA), CAPS, and Still’s disease [21]. In adult, adolescent, and pediatric patients aged 8 months or older affected by CAPS, ANA is administered at the starting dose of 1 to 2 mg/kg/day by subcutaneous (s.c.) injection. For the maintenance of response, a regimen of 1 to 2 mg/kg/day is indicated in the case of a milder disease, whereas in more severe cases, the usual maintenance dose is 3 to 4 mg/kg/day, which can be adjusted to a maximum of 8 mg/kg/day [21]. The absolute bioavailability of ANA after 70 mg s.c. injection in healthy subjects is around 95%. In RA patients, maximum plasma concentrations of ANA have been reported at 3 to 7 h after s.c. administration (1 to 2 mg/kg), whereas the half-life ranges from 4 to 6 h. The clearance of ANA is mainly mediated by the kidney, and increases along with creatinine clearance [21]. In children with CAPS, the pharmacokinetics of ANA is significantly influenced by body weight [22]. On the other hand, CANA is a human monoclonal antibody that specifically binds IL-1β, blocking its interaction with IL-1 receptor and preventing the consequent inflammatory response [23]. CANA (Ilaris^®^) is currently approved for the treatment of periodic fever syndromes in adults, adolescents, and children aged at least 2 years, including CAPS, TRAPS, FMF, and HIDS/MKD, as well as in the treatment of Still’s disease and gouty arthritis [23]. The recommended starting dose of CANA in adults, adolescents, and children aged 2 years (and older) is 150 mg for patients with body weight > 40 kg and 2 mg/kg for patients with body weight ≥ 7.5 kg and ≤ 40 kg. CANA is administered every four weeks as a single dose via s.c. injection. In CAPS children aged 2 to 4 years with body weight ≥ 7.5 kg and in adolescents and children older than 4 years with body weight between 7.5 and 15 kg, the starting dose of CANA is 4 mg/kg. In patients with body weight between 15 kg and 40 kg, the indicated starting dose is 2 mg/kg, whereas in patients weighing more than 40 kg CANA should be initially administered at the dose of 150 mg [23]. CANA should be administered every 8 weeks, as a single dose via s.c. injection. Maintenance dose should be defined based on the initial response [23]. In adults, the peak of serum CANA concentration (Cmax) occurs approximately 7 days after a single s.c. administration of 150 mg, whereas the mean half-life is around 26 days. The absolute bioavailability of CANA is estimated to be 66%. Body weight significantly influences both CANA distribution and elimination [23]. In very young children, a modest increase in the turnover rate of IL-1β has been observed. In pediatric patients, no age-related variations of CANA clearance and volume of distribution can be found after correction for body weight [24]. A schematic representation of IL-1 inhibition with CANA and ANA is depicted in Figure 1.

## 3. Cryopyrin-Associated Periodic Syndrome

CAPS includes a spectrum of apparently distinct inflammatory disorders of increasing severity: Familial cold autoinflammatory syndrome (FCAS), Muckle-Wells syndrome (MWS), and chronic infantile neurological, cutaneous, articular (CINCA) syndrome/neonatal-onset multisystem inflammatory disease (NOMID), all caused by mutations in *NLRP3*, the gene encoding cryopyrin, a component of the IL-1 inflammasome that regulates the production of IL-1β [25]. Therapeutic strategies specifically aimed at blocking IL-1 have been widely evaluated in CAPS patients (Table 1). Namely, the efficacy and safety profile of CANA in CAPS have been extensively assessed, both in clinical trials as well as in the real clinical practice. In a 48-week double-blind placebo-controlled randomized withdrawal study, the use of CANA (administered s.c. at the dose of 150 mg or 2 mg/kg for patients weighing 40 kg or less) every 8 weeks was evaluated in a cohort of 35 CAPS patients [26]. Namely, 4 patients were aged 4 to 16 years, whereas the remaining 31 patients were aged 17 to 75 years. Of them, 26 had history of a previous anti-IL1 treatment. A single dose of CANA accounted for complete response in 34 patients (97%); of note, CAPS symptoms significantly improved within 24 h in patients who had a response. Regarding patients entering the double-blind phase, all the 15 patients continuing CANA treatment remained in remission, whereas 81% of patients (13/16) in the placebo group had a disease flare, after a median time of 100 days from the start of placebo. As for safety, the proportion of patients experiencing at least one adverse event ranged from 77% in the third phase of the study to 100% in the second phase. Interestingly, during the same phase, adverse events were also recorded in 88% of patients treated with placebo. Most frequent adverse events included nasopharyngitis, rhinitis, diarrhea, nausea, influenza, bronchitis, headache, and vertigo. Overall, 9 patients experienced serious adverse events. There were no reports of severe injection-site reactions. Furthermore, no safety issues emerged regarding blood monitoring and urinalysis. In a phase II open-label study on 7 pediatric patients with CAPS (5 children with MWS and 2 adolescents with NOMID) [27], CANA (2 mg/kg s.c. for patients weighing ≤ 40 kg or 150 mg s.c. for patients weighing > 40 kg) led to a complete response within 7 days after the first dose in all cases. According to physicians’ assessments, a relevant improvement in symptoms occurred within 24 h after the first dose. Six patients were retreated on relapse, and 4 achieved a second complete response within 7 days following retreatment. CANA was generally well tolerated. Only one severe adverse event (vertigo) was reported. Most frequent adverse events were upper respiratory tract infection (*n* = 5), rash (*n* = 4), pharyngitis (*n* = 3), nasopharyngitis (*n* = 3), and vomiting (*n* = 3). In another open-label multicentre phase III study conducted on 109 CANA-naïve adult and pediatric patients and 57 patients with previous history of CANA treatment, CANA was administered at the dose of 150 mg or 2 mg/kg every 8 weeks for up to 2 years [28]. Among CANA-naïve patients, complete response was achieved in 85 cases (78%), 79 of which occurred within the first 8 days of treatment. The other 23 patients who did not achieve complete response showed variable disease improvement. Data of the relapse assessment were available for 141 patients; 90% of them did not relapse, whereas 14 had a clinical relapse on at least one occasion. Overall, the median duration of treatment was 414 days (29–687 days) for the entire cohort, and 290 days (29–625 days) for pediatric patients. In order to control disease, higher doses were required in pediatric cases (≤ 40 kg) compared with adults, and in patients with NOMID compared with other phenotypes. Overall, 40 patients needed dose (or dose frequency) adjustments to control the disease. Overall, 90.4% of patients (*n* = 150) experienced at least one adverse event. Most frequent adverse events included headache (*n* = 34), rhinitis (*n* = 27), arthralgia (*n* = 24), bronchitis (*n* = 18), diarrhea (*n* = 18), and upper respiratory tract infections (*n* = 17). Eighteen patients experienced at least one severe adverse event. In the pediatric cohort, six serious adverse events were reported, related to tonsillitis (*n* = 3), severe intra-abdominal abscess following appendicitis, severe bronchitis, and pneumonia. In a double-blind placebo-controlled randomized withdrawal study by Koné-Paut et al. [29], 35 CAPS patients (of whom 5 were pediatric) received CANA 150 mg s.c. every 8 weeks. According to the study protocol, a double-blind placebo-controlled withdrawal phase was performed from week 9 to 24, whereas in the open-label phase from week 24 to 48 all patients resumed CANA. On day 8, 89% of patients had minimal or no disease activity. By day 8, clinical response was associated with a decrease of inflammatory markers, and considerable improvement in all 36-item Short-Form Health Survey (SF-36) domain scores. Response was sustained in patients treated with 8-weekly CANA, whereas it was rapidly lost during the placebo-controlled phase in patients receiving placebo and regained following CANA resumption. The 48-week treatment with CANA was generally well tolerated. Only two patients experienced serious adverse events. Namely, one patient had recurrent antibiotic-resistant lower urinary tract infection and sepsis, which required CANA discontinuation; vertigo and increased intraocular pressure, acute glaucoma, and unilateral blindness (complications of CAPS) were observed in the second patient. In an open-label study on 19 Japanese patients aged 2 to 48 years affected by NOMID (*n* = 12) or MWS (*n* = 7), CANA was administered every 8 weeks for 24 weeks, at the dose of 150 mg s.c. or 2 mg/kg for patients with a body weight over and under 40 kg, respectively [30]. Complete response was achieved in 18 out of 19 patients, though with dose and/or frequency adjustments. At day 24, relapse occurred in four patients, whereas one patient discontinued CANA before week 24. AID activity scores and inflammatory markers significantly decreased following CANA treatment. Namely, mean C-reactive protein levels decreased by 2.94 ± 2.99 mg/dL, dropping to 1.19 mg/dL at the end of the study compared to 4.52 mg/dL at baseline. A similar trend was observed for serum amyloid-A (SAA) levels. Interestingly, anti-CANA antibodies were detected in 3/19 patients, but the presence of these antibodies was not confirmed in subsequent evaluations. As for the CANA safety profile, 18 patients (95%) experienced one or more adverse events. Specifically, most common adverse events included nasopharyngitis (*n* = 7), gastroenteritis (*n* = 6), upper respiratory tract infection (*n* = 3), and rhinorrhea (*n* = 3). Most adverse events were mild, and only 3 were considered of moderate severity. Severe adverse events related to diffuse vasculitis (*n* = 1) and pneumonia (*n* = 1) were also reported. Higher CANA doses (> 150 mg or 2 mg/kg every 8 weeks) did not appear to be associated with a differential safety profile. In the extension phase of this trial [31], both the efficacy and safety of CANA were evaluated over a 22 month-period. After 48 weeks of treatment, as well as at the end of the study period, all patients had a complete response. All patients experienced at least one adverse event during the study, with the most common event being upper respiratory tract infections (*n* = 14). Serious adverse events were recorded in five patients, and included multiple infections, pneumonia, sinoatrial block, headache, asthma, and appendicitis. No permanent discontinuation of CANA due to adverse events was reported. Regarding ANA, a 5-year prospective open-label cohort study evaluated the safety profile of ANA in 43 CAPS patients (of whom 36 were children) [32]. The ANA starting dose ranged from 0.5 to 1.5 mg/kg/day, but was adjusted to 1.5 to 2.5 mg/kg/day during the follow-up period. The number of adverse events reported during the 5-year study period was 1233, giving an overall reporting rate of 7.7 events per patient per year. Most frequent adverse events included headache (*n* = 21), arthralgia (*n* = 18), and upper respiratory tract infections (*n* = 17). Serious adverse events occurred in 14 patients, mostly within the first year of treatment and mostly related to infections. There were no deaths and all adverse events resolved during the study period. 

We report below, distinctly, the most severe forms of CAPS, including CINCA/NOMID and MWS, focusing on the main studies in which ANA and CANA were employed.

CINCA/NOMID represents the most severe form of CAPS and is characterized by the triad of cutaneous urticarial-like rash, arthropathy, and central nervous system (CNS) involvement, in association with typical dysmorphic features, including frontal bossing and saddle back nose. CNS manifestations encompass chronic aseptic meningitis leading to brain atrophy and sensorineural hearing loss. Anti-IL-1 treatment represents the standard therapy for this condition. A trial by Goldbach-Mansky et al. evaluated both the efficacy and safety of ANA (1 to 2 mg/kg s.c.) in 18 NOMID patients [33]. All patients had a prompt clinical response to ANA. In detail, rash and conjunctivitis disappeared within three days and all inflammatory markers, including SAA, rapidly dropped. After 3 months of treatment, 11 patients underwent a withdrawal phase of a maximum of 7 days. Disease flare occurred in all except one patient, after a median time of 5 days (2.5 to 7 days). ANA resumption was associated with a rapid response, and improvements were maintained over the 6 month follow-up. As for specific CNS response, ANA treatment was associated with a significant decrease from 0.5 to 0.1 (*p* < 0.001) in the median daily headache scores (classified from 0 to 4 for increasing severity). In 12 patients in whom cerebrospinal fluid was evaluated, intracranial pressure, protein levels, and white-cell counts also decreased significantly. Furthermore, brain magnetic resonance imaging showed a relevant improvement in cochlear and leptomeningeal lesions as compared with baseline. Adverse events reported during ANA treatment included upper respiratory infections (*n* = 15), urinary tract infections (*n* = 2), and dehydration from nonbacterial diarrhea requiring hospitalization (*n* = 1). Localized, injection-site erythematous reactions were reported in eight cases. However, none of the patients discontinued drug treatment. The use of ANA in NOMID is further supported by evidence coming from four observational studies [43,44,45] as well as from different isolated experiences (see details in Table 1).

In a 24-month open-label phase I/II study [34], six patients aged 11 to 34 years were treated with CANA 150 mg (or 2 mg/kg in patients ≤ 40 kg) or 300 mg (or 4 mg/kg) with escalation up to 600 mg (or 8 mg/kg) every 4 weeks, after discontinuation of previous ANA treatment. CANA led to a significant improvement in all patients, with four patients having inflammatory remission at month 6. CNS remission was not achieved by any of the six patients. However, five patients had a significant improvement in their headache diary scores, and the other patient had a normal CSF leucocyte count, albeit showing a persistent headache. Overall, CANA was well tolerated. Only one severe adverse event related to a methicillin-resistant *Staphylococcus aureus* abscess was recorded. Twelve infection-related adverse events occurred in six patients.

MWS is a rare autosomal dominant disease belonging to the family of CAPS: Its manifestations include urticaria-like rashes, arthralgia, progressive sensorineural deafness, episodic fever, and renal amyloidosis. IL-1 inhibitors have been shown to be revolutionary in MWS [46]. The use of ANA in MWS was evaluated in a single-center observational study on 12 patients (5 children and 7 adults) with severe MWS [47] (see Table 1). After 2 weeks of treatment, a significant decrease in disease activity was reported, leading to significant improvement of organ manifestations as well as improved inflammatory parameters. Treatment was well tolerated, and no severe adverse events were reported. In another single-center open-label prospective observational study on patients diagnosed with active MWS between 2004 and 2008, ANA was started in five pediatric and seven adult patients, whereas CANA was initiated in six children and eight adults [48]. Both treatments led to a significant reduction of disease activity and inflammatory parameters. After a mean time of 12 months (range 5 to 14 months) for ANA and 11 months (6 to 15 months) for CANA, disease remission was achieved in 75% and 93% of patients, respectively. No detectable difference in treatment efficacy was found when comparing anti-IL-1 naïve versus rollover patients (i.e., treated with CANA after ANA discontinuation). In the ANA cohort, no serious adverse events were observed. Mild adverse events included injection-site reactions in five patients and mild upper respiratory infections in four patients, respectively. In the CANA cohort, vertigo occurred in one patient and required hospitalization. No injection-site reactions were observed. Other adverse events included mild upper respiratory tract symptoms in four patients and transient headache in two patients. The use of ANA in MWS is further supported by evidence deriving from five case reports [49,50,51,52,53].

## 4. Tumor Necrosis Factor Receptor-Associated Periodic Syndrome

TRAPS is the most frequent autosomal dominant autoinflammatory disorder, which is caused by mutations in the *TNFRSF1A* gene, encoding the 55-kD type-1 receptor of tumor necrosis factor (TNF)-α [54]. The average age at disease onset is around 3 years; however, adult onset has been described up to the sixth decade of life. This disease is clinically characterized by recurrent episodes of long-lasting fever and inflammation involving different organs, such as the skin, gastrointestinal tract, serous membranes, joints, muscles, and eyes. Inflammatory attacks are initially responsive to corticosteroids, but the progressive loss of response and recurrence of uncontrolled attacks is further associated with the development of secondary amyloidosis [55]. As IL-1-mediated inflammation is directly involved in the pathogenesis of this syndrome, anti-IL-1 treatments are gaining a relevant role in the management of this disorder (Table 1) [56,57]. Namely, Gattorno et al. evaluated the use of ANA in four children and one adult with TRAPS [37]: All patients had a rapid response to ANA, with a disappearance of symptoms and normalization of inflammatory parameters, including SAA. According to the study protocol, ANA was discontinued after 15 days of treatment. In all cases, a disease relapse was observed, within a mean time of 6 days (range, 3 to 8 days), whereas reintroduction of ANA was associated with a regain of disease control. Over a mean follow-up of 11 months, no fever episodes as well as no disease-related clinical manifestations were observed. The only adverse events reported were cutaneous reactions, including rash, pain, and itching, all occurring during the first week of treatment. Use of CANA in TRAPS has been evaluated in an open-label phase II study on 20 patients aged 7 to 78 years, with active recurrent or chronic TRAPS [38]. CANA, at the dose of 150 mg every 4 weeks for 4 months or 2 mg/kg for patients weighing 40 kg or less, induced clinical remission (Physician’s Global Assessment score ≤ 1) and full or partial serological remission within day 15 in 95% of patients (*n* = 19). Responses to CANA occurred rapidly, with a median time to clinical remission of 4 days (3–8 days). Furthermore, a significant improvement in the quality of life was also reported. According to the study protocol, CAN was withdrawn after 4 months of treatment: All patients relapsed following CANA discontinuation after a median time of 91.5 days (range of 65 to 117 days). However, CANA was well tolerated. All patients reported at least one adverse event, and most common events were nasopharyngitis (*n* = 12), abdominal pain (*n* = 11), headache (*n* = 11), and oropharyngeal pain (*n* = 10). Serious adverse events were reported in seven patients, and included pericarditis, abdominal pain, diarrhea, intestinal obstruction, vomiting, upper respiratory tract infection, meniscus injury, hypertriglyceridemia, and hyperkalemia. No significant changes in laboratory, clinical, and vital parameters were reported, and no patients developed anti-drug antibodies.

## 5. Familial Mediterranean Fever

FMF is the most frequent autosomal recessive autoinflammatory disorder, characterized by self-limited episodes of fever and polyserositis, which may also lead to long-term complications, such as renal amyloidosis [58,59]. Although its pathogenesis is not fully understood, the *MEFV* gene encodes mutant pyrins, crucial players in the regulation of innate immunity, which lead to uncontrolled production of IL-1 [59]. Use of CANA in colchicine-resistant FMF (crFMF) is supported by three clinical trials (Table 1). In the 6-month open-label, single-arm pilot study by Brik et al. [40], seven children with crFMF were treated with subcutaneous injections of CANA at the dose of 2 mg/kg (maximum 150 mg), 4 weeks apart. Six patients experienced a reduction of 76% to 100% of FMF attacks, and three did not experience any attacks during the treatment phase. After the last CANA injection, five participants developed an attack, after a median time of 25 days (range of 5 to 34 days). Overall, 11 adverse events were reported in four patients, including two cases of infections. All adverse events were mild, except a moderate streptococcal throat infection. No significant laboratory abnormalities and formation of neutralizing anti-CANA antibodies were reported. In another open-label trial on nine adolescents and adults with crFMF [41], CANA accounted for a reduction of 50% or more in attack frequency in all treated patients, with only one patient experiencing an attack during the treatment period. Furthermore, following CANA administration, a significant improvement was observed in both the physical and mental component assessed by SF-36. Following the last CANA injection, five patients had an attack, after a median time of 71 days (range of 31 to 78 days). Eight patients (89%) experienced one or more adverse events. Namely, the most frequent adverse events were headache (*n* = 4), and respiratory tract infections. Other adverse events included, anxiety, hidradenitis, pruritus, tooth infection, and vomiting, all reported in one case each. Effectiveness and safety of CANA in crFMF is further supported by two long-term retrospective observational studies on 15 children [60] and 14 adolescent or adult patients [61], respectively. On the other hand, the use of ANA in FMF is mainly supported by observational evidence. In a study by Başaran et al. [62], eight children and adolescents with crFMF were treated with ANA (1 mg/kg/day to 3 mg/kg/day). A switch to CANA was required in four cases, due to noncompliance to daily injections in three cases and clinical and laboratory worsening in one case. Overall, the use of anti-IL-1 drugs was beneficial to all patients, both in terms of a reduction of attack frequency and normalization of inflammatory parameters. No severe adverse events were reported, and only one patient experienced a local injection-site erythema with ANA. 

Yet, in another retrospective study by Cetin et al. [63] conducted on 20 patients with crFMF, 4 pediatric patients were treated with ANA or CAN (in two cases each). In two children treated with ANA, the number of monthly attacks decreased from 4 to 0 in one case and from 1 to 0 in the other one. ANA was continued for 12 and 7 months, respectively, and was still ongoing at the end of the study. Similarly, CANA also accounted for a significant reduction both in the rate of monthly and annual attacks. No adverse skin or allergic events was reported. Biopsy-proven amyloidosis was present in one child treated with CANA, with a significant decrease of urinary protein excretion (from 25.6 mg/m^2^/h to 12 mg/m^2^/h before versus after CANA treatment, respectively). In the retrospective study by Özçakar et al. [64], three pediatric patients suffering from crFMF were treated with ANA and one case with CAN. Three of them (two treated with ANA and one with CANA) had concomitant FMF-related amyloidosis. Among the three patients treated with ANA, the occurrence of attacks was significantly reduced. However, for the two patients with amyloidosis, one needed renal transplantation and the other had end-stage renal disease. In the patient treated with CANA, the frequency of disease attacks was significantly reduced (from 24 attacks/year to 0), and partial remission of nephrotic syndrome was achieved. None of these patients experienced drug-related adverse events.

## 6. Hyperimmunoglobulin D Syndrome/Mevalonate Kinase Deficiency

HIDS/MKD is an autosomal recessive disorder caused by mutations in the gene encoding the enzyme mevalonate kinase, directly involved in cholesterol and isoprenoid biosynthesis. This autoinflammatory disease usually starts in the first year of life and is characterized by lifelong recurrent fever episodes (every 4 to 6 weeks), typically lasting from 3 to 7 days [65,66]. The clinical signs range from the milder HIDS to its most severe expression, named “mevalonic aciduria”. The most frequent symptoms during febrile attacks, sometimes precipitated by vaccinations, infections, emotional stress, trauma, or surgery, are abdominal pain, diarrhea, vomiting, arthralgia, lymphadenopathy, heterogeneous skin lesions, and aphthous ulcers [4]. An increase in mevalonic acid and activation of small GTPases result in IL-1 overexpression via caspase-1 activation. Therefore, short-term IL-1 blockade may be effective for stopping inflammatory attacks [2]. To date, two clinical trials have evaluated the use of the IL-1 inhibitor, CANA, in this condition, whereas no controlled clinical trial on ANA has been conducted (Table 1). Namely, in the open-label phase II study by Arostegui et al. [42], CANA was administered subcutaneously at the dose of 300 mg (or 4 mg/kg for patients weighing ≤ 40 kg) every 6 weeks to six pediatric patients and three adults with active HIDS. In this cohort, the first CANA injection led to good or excellent control of the disease, with a median time to disease resolution of 3 days. Furthermore, CANA accounted for a significant reduction in the severity or disappearance of HIDS features, including fever, lymphadenopathy, abdominal pain, and aphthous ulcers. According to the study protocol, CANA was discontinued after 6 months. Seven out of nine patients experienced a relapse following CANA discontinuation, the median time to relapse being 110 days (range of 62 to 196 days). As for its safety profile, all nine patients experienced at least one adverse event during the study period, albeit only one was judged as drug-related (i.e., non-serious fungal vaginitis). Overall, 98 adverse events (of whom 14 serious) were observed. Most frequent non-serious adverse events were related to infections and required systemic antibiotics in most cases. As for serious adverse events, 8 out of 14 events included acute peritonitis, anemia, bacteremia due to *Streptococcus pneumoniae*, gastrointestinal bleeding, hypertensive crisis, pneumonia, and severe anemia, which occurred in the same patient. Another patient was hospitalized due to hidradenitis suppurativa, and one patient developed cellulitis in the left arm. No death was reported, and no patient required drug discontinuation because of adverse events. Furthermore, no relevant change in clinical laboratory parameters and vital signs was observed. 

Use of ANA in the treatment of pediatric HIDS/MKD is supported only by observational evidence. Specifically, two studies evaluated both the efficacy and safety of ANA for this condition. In the prospective observational study by Bodar et al. [67], 11 patients with HIDS (4 pediatric patients aged 5 to 17) were treated with either continuous (*n* = 3) or on-demand (*n* = 8) ANA (starting at first symptoms of an attack, 100 mg/day or 1 mg/kg/day for 5 to 7 days). In the two pediatric patients with mevalonic aciduria, continuous ANA treatment induced partial remission only in one case, and no response in the other one. Among the other nine patients, continuous ANA treatment induced complete remission, but was further discontinued for safety reasons. Among the nine patients with on-demand therapy, ANA induced a clinical response in 8 out of 12 attacks, but did not impact on its frequency. No major adverse events were observed; local injection-site reactions as well as mild upper respiratory tract infections were the only adverse events. Of note, treatment discontinuation was required in one patient. In the cohort of 103 adult and pediatric HIDS patients investigated in the observational study by van der Hilst et al., the use of ANA was reported in 11 cases: Among them, four achieved a good response to treatment, three a partial response, whereas the remaining four had no response to therapy [68]. Use of both ANA and CANA has been further evaluated in an observational study by Galeotti et al. [69]: Eleven French adult and pediatric patients were treated with either ANA (*n* = 9) or CANA (*n* = 6, of whom four following ANA treatment), reaching a complete and partial remission in four and seven cases, respectively. Anti-IL-1 treatment was also associated with a decrease in a 12-item clinical score, in the number of days with fever during attacks as well as in the level of inflammatory parameters. Both drugs were well tolerated. During ANA treatment, four patients experienced injection-site reactions, whereas shivers and hypothermia after the first injection and bacterial pneumonia were reported in one patient each. As for CANA, adverse events included injection-site reaction (*n* = 1), recurrent pharyngitis (*n* = 1), and transient hepatitis (*n* = 2, one of whom was without confirmation of viral or autoimmune etiology). No alterations in hematological and urinary parameters were reported. Yet, in a national Japanese survey conducted on 10 pediatric patients suffering from MKD, the use of anti-IL-1 treatment was reported in two patients [70]: In both patients, initial ANA treatment accounted for partial response, whereas a switch to CANA led to a complete response. During anti-IL-1 treatment, transaminase elevation and arthritis were reported in one patient each. 

## 7. Additional Evidence on IL-1 Inhibition in Autoinflammatory Diseases

Beside the above summarized evidence for specific AIDs, the use of anti-IL-1 has been evaluated also in mixed cohorts of patients with different conditions. 

A retrospective chart review by Ozen et al. evaluated the treatment pattern of 134 patients with FMF (*n* = 49), TRAPS (*n* = 47), or HIDS/MKD (*n* = 38), highlighting the central role of anti-IL-1 agents in the management of these conditions [71]. Similarly, data derived from the Eurofever Registry and related to 496 patients with FMF, CAPS, TRAPS, MKD, pyogenic arthritis-pyoderma gangrenosum-acne (PAPA) syndrome, deficiency of IL-1 receptor antagonist (DIRA), NLRP12-related autoinflammatory disorder, and periodic fever-aphthosis-pharyngitis-adenitis (PFAPA) syndrome pointed out the key-role of IL-1 blockade for DIRA and CAPS, as well as for cases of poorly controlled MKD, TRAPS, PAPA, and crFMF [72]. According to an Italian study aimed at evaluating the use of IL-1 inhibitors among 475 patients (of whom 111 were aged 16 or less), 86% and 56% of all treatments with ANA and CANA, respectively, were mainly related to adult onset Still’s disease (18.5%), SJIA (13.5%), Behçet’s disease (9.7%), FMF (7.6%), idiopathic recurrent acute pericarditis (5.6%), and TRAPS (5.0%) in the ANA group, and to Behçet’s disease (14.3%), TRAPS (13.3%), FMF (5.7%), and HIDS (3.8%) in the CANA one [73].

Efficacy and safety of ANA in patients with AIDs is further supported by the result of a French nationwide survey on 189 patients [74]. On the other hand, damage caused by amyloid deposits in AIDs seems not to improve with anti-IL 1 treatment [75]. 

In a recent clinical trial by De Benedetti et al. [36], 63 patients with crFMF (29 were children), 72 with MKD (54 children), and 46 with TRAPS (27 children) were randomized to receive CANA 150 mg s.c. or placebo every 4 weeks, with an add-on injection of 150 mg of CANA in the case of no flare resolution. At week 16, the proportion of patients with complete response was significantly higher in the CANA group if compared with the placebo group: Sixty-one percent versus 6% for patients with crFMF (*p* < 0.001), 35% versus 6% for MKD (*p* = 0.003), and 45% versus 8% for TRAPS (*p* = 0.006). Considering also patients who required an increase in the CANA dose, a complete response was achieved in 71% of patients with crFMF, in 57% of patients with MKD, and in 73% of those with TRAPS. After 16 weeks, disease control was maintained in 46%, 23%, and 53% of patients with crFMF, MKD, and TRAPS, respectively. Numbers of adverse events observed during CANA treatment were 332, 613, and 265 among patients with crFMF, MKD, and TRAPS, respectively. Namely, numbers of adverse events related to infections were 79, 160, and 58 in the three disease groups. As for events unrelated to infections, most frequent adverse events included abdominal pain, headache, arthralgia, and injection-site reactions. This study led to market authorization of CANA for the three conditions. 

## 8. Systemic Juvenile Idiopathic Arthritis

SJIA is an inflammatory disease associated with dysregulation of the innate immune system [7,9]. The disease is characterized by fever, lymphadenopathy, arthritis, rash, and serositis. Furthermore, complications of SJIA include invalidating arthritis and MAS, a condition characterized by unremitting high fever, pancytopenia, hepatosplenomegaly, hepatic dysfunction, encephalopathy, coagulation abnormalities, and increased levels of ferritin. As IL-1 and IL-6 have been shown to play a primary role in the pathogenesis of SJIA, anti-IL-1 treatments as well as anti-IL-6 drugs represent promising therapeutic strategies for the control of this disease [7,9]. The use of IL-1 inhibitors, ANA and CANA, in SJIA has been extensively evaluated, both in clinical trials and observational studies (Table 2). In a study by Gattorno et al. [76], ANA (at the starting dosage of 1 mg/kg/day, for a maximum of 100 mg) was administered to 22 patients aged 1 to 19 years. Within the first week of treatment, two distinct patterns of response to ANA could be distinguished. One group of 10 patients achieved prompt improvement of systemic and articular manifestations as well as improved inflammatory parameters, maintaining complete disease control during a mean follow-up of 1.36 years (range of 0.3 to 2.59 years). On the other hand, a second group of 11 patients experienced a variable response to ANA, with an improvement soon after treatment began, but with a general tendency towards relapses, particularly at the articular level. These two clusters appeared to be characterized by different clinical features, in particular patients with complete response had a significantly lower number of active joints and an increased absolute neutrophil count. 

Another multicentre randomized double-blind placebo-controlled trial compared the efficacy of ANA (2 mg/kg s.c. daily, maximum 100 mg) versus placebo in 24 SJIA patients aged 8.5 ± 4.5 years [82]. After 1 month of treatment, response (defined as a 30% improvement of the pediatric American College of Rheumatology (ACR) criteria for JIA, resolution of systemic symptoms, and a decrease of at least 50% of inflammatory parameters compared with baseline) was achieved in 8 out of 12 patients treated with ANA and only in 1 patient receiving placebo (*p* = 0.003). Ten patients were switched from placebo to ANA; of them, nine achieved response within month 2. Fourteen adverse events were recorded in the ANA group and 13 in the placebo group. No serious adverse event was reported. Namely, in the ANA group, adverse events, including injection-site pain (*n* = 8), post-injection erythema (*n* = 3), and infections involving gastrointestinal and upper respiratory tract (*n* = 2), were recorded. 

In a multicenter double-blind trial on 50 patients aged 3 to 17 years with polyarticular-course JIA (11 of whom had systemic onset), randomized to ANA (1 mg/kg/day, for a maximum of 100 mg) or placebo [80], the three most common reported adverse events were injection-site reactions (12% in each group), upper respiratory infections (16% versus 20% in the ANA and placebo groups, respectively), and headache (24% versus 4%). No case of adverse event-related discontinuation of the blinded phase was observed. In the subsequent extension phase of ANA treatment, the most common adverse events reported were arthralgia (23%), fever (21%), and abdominal pain (16%), with three patients (7%) requiring discontinuation of ANA because of safety issues. 

In another pilot study evaluating different therapeutic strategies for treatment of SJIA [81], 12 out of 30 patients received IL-1 inhibitors (ANA as initial treatment, eventually switched to CANA). Of them, two patients needed to add methotrexate, whereas two needed to switch to an IL-6 inhibitor. Overall, clinical disease inactivity was reached in 42% of patients treated with IL-1 inhibitors. There were four serious adverse events: Two resulted in hospitalization for intravenous antimicrobial therapy (cellulitis in a child taking CANA and glucocorticoids, and varicella in a child taking ANA), one hospitalization for appendicitis and appendectomy in a child on methotrexate and glucocorticoid, and one for MAS in a child on tocilizumab. As for CANA, its efficacy and safety in SJIA has been extensively evaluated in a pooled analysis of data coming from four trials [78,83,84,85]. CANA (mostly administered at the dose of 4 mg/kg every 4 weeks) was given to a total population of 233 children, 60 young adolescents and 31 older adolescents or young adults. Within day 15 of treatment, at least 50% of patients in each age group had absence of fever as well as ≥ 70% improvement according to the adapted ACR pediatric response criteria. Responses were stable and maintained or improved over the 85 days of follow-up. Similarly, clinical and laboratory findings also markedly improved in all age groups. Regarding safety, adverse events were reported in 86.7% to 88.3% of patients in the different age groups, with 11% to 19% of patients who experienced adverse events leading to treatment discontinuation. In all age groups, most common adverse events were infections (70–76%), gastrointestinal disorders (52–58%), and musculoskeletal or connective tissue disorders (51–55%). Other adverse events included disorders of the skin, subcutaneous tissue, and respiratory tract. Serious adverse events were reported in 29% to 42% of patients, and included JIA reactivation, MAS, gastroenteritis, and *Cytomegalovirus* infection. Beside the above mentioned evidence from clinical trials, the use of ANA and CANA in SJIA is also supported by growing evidence coming from observational studies [79,86,87,88,89,90,91,92,93].

## 9. Kawasaki Disease

KD is an acute vasculitis of unknown etiology, which is typically observed in the pediatric age. If untreated, patients with KD are at significantly higher risk of developing coronary artery abnormalities, thromboembolic occlusions, and myocardial infarction, with subsequent increased risk of mortality [94]. Many shortcomings still exist in studies aimed at defining the etiology of KD, though different levels of evidence support the hypothesis that it is a complex disease with a unique pathogenesis [95]. Intravenous immunoglobulins (IVIG) in association with aspirin represent the main treatment for KD and their administration within the first 10 days following fever onset has been associated with a 5-fold reduction in the risk of coronary artery aneurysms [96]. 

The extent of acute phase response and a younger age at onset may be related to patients’ responsiveness to IVIG [97]. In particular, 10% to 15% of patients develop resistance to this treatment: The prediction of IVIG resistance is a crucial issue for managing these children, as recognizing high-risk patients should allow the administration of an intensified initial treatment in combination with IVIG, and prevent coronary injuries [98]. Limited and local experiences suggest the possible benefit of IL-1 inhibition in children with KD [99,100,101,102,103,104]. A retrospective case series by Koné-Paut et al. [105] evaluated the use of ANA (2 to 8 mg/kg) in 11 children with KD aged 4 months to 9 years refractory to standard treatment. Specifically, the main reasons for starting ANA were persistent inflammation, progression of coronary dilations, and severe myocarditis with cardiac failure. ANA proved to be effective in controlling KD. Namely, all patients had fever resolution and a decrease of inflammatory parameters. Furthermore, 10 out of 11 patients had a decrease in coronary artery dilations. The other patient died suddenly due to massive pericardial effusion secondary to giant aneurysm rupture while on anticoagulant treatment. To date, two trials with ANA in children with Kawasaki disease are ongoing (NCT02179853 and NCT02390596).

## 10. Conclusions

Recent evidence from both observational studies and clinical trials have clarified the efficacy of ANA and CANA in the main AIDs, also revealing a good safety profile with minor concerns regarding tolerability. In particular, the major treatment-related side effects of ANA are skin reactions at the injection-site. This high rate of injection-site reactions can become so irritating for pediatric patients that they require treatment withdrawal. In this regard, convincing patients, especially children, to continue therapy can be challenging. Reactions can be mitigated by the application of topical hydrocortisone or anti-histamine cream, but it may not be enough [106]. On the other hand, the overall safety of CANA has shown an excellent tolerability [107], as highlighted by very few discontinuation rates and few injection-site reactions. However, a slightly increased rate of non-serious infections related to the upper respiratory tract has been observed [26]. Although these two anti-IL1 agents represent the most effective treatments available in AIDs and also a promising tool in refractory KD, the development of novel pharmacological formulations that further reduce side effects in pediatric sceneries is expected.

## Figures and Tables

**Table 1:** Main clinical trials evaluating the use of Anakinra (ANA) and Canakinumab (CAN) for the treatment of cryopyrin-associated periodic syndrome (CAPS), tumor necrosis factor receptor-associated periodic syndrome (TRAPS), familial Mediterranean fever (FMF), and hyperimmunoglobulin D syndrome (HIDS)/mevalonate kinase deficiency (MKD) in the pediatric population.

Authors	Title	Study Design	Population	Drug
**Cryopyrin-Associated Periodic Syndrome (Including CINCA/NOMID and MWS)**				
Goldbach-Mansky et al., 2006 [33]	Neonatal-onset multisystem inflammatory disease responsive to interleukin-1beta inhibition.	Clinical trial	Pediatric + adults (total *n* = 18)	ANA (1 to 2 mg/kg/day s.c.)
Goldbach-Mansky et al., 2006 [33]	Neonatal-onset multisystem inflammatory disease responsive to interleukin-1beta inhibition.	Clinical trial	Pediatric (*n* = 15) + adults (*n* = 3)	ANA (1 to 2 mg/kg/day s.c.)
Imagawa et al., 2013 [30]	Safety and efficacy of Canakinumab in Japanese patients with phenotypes of cryopyrin-associated periodic syndrome as established in the first open-label, phase-3 pivotal study (24-week results).	Phase-3 pivotal study	Pediatric + adults (total *n* = 19)	CANA (150 mg s.c. (or 2 mg/kg for patients with a body weight ≤ 40 kg) every 8 weeks for 24 weeks)
Koné-Paut et al., 2011 [29]	Sustained remission of symptoms and improved health-related quality of life in patients with cryopyrin-associated periodic syndrome treated with Canakinumab: Results of a double-blind placebo-controlled randomized withdrawal study.	Double-blind placebo-controlled randomized withdrawal study	Pediatric + adults (total *n* = 35)	CANA (150 mg s.c. at 8-week intervals)
Kuemmerle-Deschner et al., 2011 [27]	Canakinumab (ACZ885, a fully human IgG1 anti-IL-1β mAb) induces sustained remission in pediatric patients with cryopyrin-associated periodic syndrome (CAPS).	Phase II, open-label study	Pediatric (*n* = 7)	CANA (2 mg/kg s.c. for patients ≤ 40 kg or 150 mg s.c. for patients > 40 kg; re-dosing upon each relapse)
Kuemmerle-Deschner et al., 2011 [28]	Two-year results from an open-label multicentre phase III study evaluating the safety and efficacy of Canakinumab in patients with cryopyrin-associated periodic syndrome across different severity phenotypes.	Open-label, multicentre, phase III study	Pediatric (*n* = 47) + adults (*n* = 119)	CANA (s.c. 150 mg or 2 mg/kg for patients ≤ 40 kg every 8 weeks for up to 2 years)
Kullenberg et al., 2016 [32]	Long-term safety profile of Anakinra in patients with severe cryopyrin-associated periodic syndrome.	Prospective, open-label, single center trial	Pediatric (*n* = 36) + adults (*n* = 7)	ANA (starting dose 0.5 to 2.5 mg/kg/day)
Lachmann et al., 2009 [26]	Use of Canakinumab in the cryopyrin-associated periodic syndrome.	Double-blind, placebo-controlled, randomized withdrawal study	Pediatric (*n* = 4) + adults (*n* = 31)	CANA (part 1: 150 mg s.c.; part 2: Either 150 mg of CANA or placebo every 8 weeks for up to 24 week)
Sibley et al., 2017 [34]	A 24-month open-label study of Canakinumab in neonatal-onset multisystem inflammatory disease.	Open-label study	Pediatric (*n* = 2) + adults (*n* = 4)	CANA (150 mg (or 2 mg/kg in patients ≤ 40 kg) or 300 mg (or 4 mg/kg) with escalation up to 600 mg (or 8 mg/kg) every 4 weeks)
Wikén et al., 2018 [35]	Development and effect of antibodies to Anakinra during treatment of severe CAPS: Sub-analysis of a long-term safety and efficacy study.	Post hoc analysis on data from a prospective, open-label, single-center, clinical cohort study	Pediatric (*n* = 36) + adults (*n* = 7)	ANA (1.0 to 2.4 mg/kg/day s.c., increased to 2.0 to 5.0 mg/kg/day according to clinical need (median 3.1 mg/kg/day))
Yokota et al., 2017 [31]	Long-term safety and efficacy of Canakinumab in cryopyrin-associated periodic syndrome: results from an open-label, phase III pivotal study in Japanese patients.	Open-label, phase III pivotal study	Pediatric + adults (*n* = 35)	CANA (2 to 8 mg/kg s.c. every 8 weeks)
**Tumor Necrosis Factor Receptor-Associated Periodic Syndrome**				
De Benedetti et al., 2018 [36]	Canakinumab for the treatment of autoinflammatory recurrent fever syndromes.	Clinical trial	Pediatric (TRAPS *n* = 27) + adult (TRAPS *n* = 19)	CANA (initial phase 150 mg/4 weeks)
Gattorno et al., 2008 [37]	Persistent efficacy of Anakinra in patients with tumor necrosis factor receptor-associated periodic syndrome.	Clinical trial	Pediatric (*n* = 4) + adult (*n* = 1)	ANA (1.5 mg/kg/day)
Gattorno et al., 2017 [38]	Canakinumab treatment for patients with active recurrent or chronic TNF receptor-associated periodic syndrome (TRAPS): An open-label, phase II study.	Open-label, phase II study	Pediatric (*n* = 6) + adults (*n* = 14)	CANA (150 mg every 4 weeks for 4 months (2 mg/kg for patients ≤ 40 kg))
Torene et al., 2017 [39]	Canakinumab reverses overexpression of inflammatory response genes in tumor necrosis factor receptor-associated periodic syndrome.	Open-label, multicentre, proof-of-concept study (gene analysis)	Pediatric (n.a.) + adults (n.a.)	CANA (150 mg every 4 weeks for 4 months)
**Familial Mediterranean Fever**				
Brik et al., 2014 [40]	Canakinumab for the treatment of children with colchicine-resistant familial Mediterranean fever: A 6-month open-label, single-arm pilot study.	Open-label, single-arm pilot study	Pediatric (*n* = 7)	CANA (2 mg/kg (maximum 150 mg); the dose was doubled to 4 mg/kg (maximum 300 mg) if an attack occurred between day 1 and day 29)
De Benedetti et al., 2018 [36]	Canakinumab for the treatment of autoinflammatory recurrent fever syndromes.	Clinical trial	Pediatric (crFMF *n* = 29) + adult (crFMF *n* = 34)	CANA (initial phase 150 mg every 4 weeks)
Gül et al., 2015 [41]	Efficacy and safety of Canakinumab in adolescents and adults with colchicine-resistant familial Mediterranean fever.	Open-label pilot study	Pediatric (n.a.) + adults (n.a.)	CANA (3 injections 150 mg s.c. injections every 4 weeks)
**Hyperimmunoglobulin D Syndrome/Mevalonate Kinase Deficiency**				
Arostegui et al., 2017 [42]	Open-label phase II study to assess the efficacy and safety of Canakinumab treatment in active hyperimmunoglobulinemia D with periodic fever syndrome.	Clinical trial	Pediatric (*n* = 6) + adults (*n* = 3)	CANA (300 mg or 4 mg/kg every 6 weeks)
De Benedetti et al., 2018 [36]	Canakinumab for the treatment of autoinflammatory recurrent fever syndromes.	Clinical trial	Pediatric (MKD *n* = 54) + adult (MKD *n* = 18)	CANA (initial phase 150 mg/4 weeks)

ANA: Anakinra; CANA: Canakinumab; n.a.: not applicable; s.c.: subcutaneous.

**Table 2:** Main clinical trials evaluating the use of Anakinra (ANA) and Canakinumab (CANA) for the treatment of systemic juvenile idiopathic arthritis (SJIA) in the pediatric population.

Systemic Juvenile Idiopathic Arthritis				
Authors	Title	Study Design	Population	Drug
Brachat et al., 2017 [77]	Early changes in gene expression and inflammatory proteins in systemic juvenile idiopathic arthritis patients on Canakinumab therapy.	Gene expression analysis (data from the two phase-3 trials evaluating CANA for SJIA)	Pediatric (n.a.)	CANA (Trial 1: Patients were randomly assigned to a single s.c. dose of CANA (4 mg/kg) or placebo. Trial 2: Open-label phase (s.c. CANA 4 mg/kg every 4 weeks for up to 32 weeks) + withdrawal phase)
Feist et al., 2018 [78]	Efficacy and safety of Canakinumab in patients with Still’s disease: Exposure-response analysis of pooled systemic juvenile idiopathic arthritis data by age groups.	Pooled results of clinical trials	Pediatric (*n* = 216) + adolescents (*n* = 56) + adult (*n* = 29)	CANA (Study 1: Single s.c. CANA at 4 mg/kg (maximum of 300 mg) or placebo; Study 2: CANA 4 mg/kg (maximum dose of 300 mg) every 4 weeks for up to 8 months + second double-blind randomized placebo-controlled phase; Study 3: S.c. CANA 4 mg/kg every 4 weeks for 12 weeks (patients had received CANA in either Study 1 or Study 2, with an additional cohort of CANA-naïve patients); Study 4: Dose-ranging study (0.5–9 mg/kg)
Gattorno et al., 2008 [76]	The pattern of response to anti-interleukin-1 treatment distinguishes two subsets of patients with systemic-onset juvenile idiopathic arthritis.	Clinical study	Pediatric (*n* = 22)	ANA (starting dosage of 1 mg/kg/day, s.c. (maximum 100 mg))
Grom et al., 2016 [79]	Rate and clinical presentation of macrophage activation syndrome in patients with systemic juvenile idiopathic arthritis treated with Canakinumab.	Pooled analysis	Pediatric (*n* = 21)	CANA (n.a.)
Ilowite et al., 2009 [80]	Anakinra in the treatment of polyarticular-course juvenile rheumatoid arthritis: Safety and preliminary efficacy results of a randomized multicenter study.	2-week open-label run-in phase	Pediatric (*n* = 86)	ANA (1 mg/kg daily, maximum 100 mg/day)
Kimura et al., 2017 [81]	Pilot study comparing the Childhood Arthritis & Rheumatology Research Alliance (CARRA) systemic juvenile idiopathic arthritis consensus treatment plans.	Pilot interventional study	Pediatric (*n* = 30; IL-1 inhibitors *n* = 12)	ANA (CANA) (median initial dose of ANA 2.93 (IQR 2–3.6))
Quartier et al., 2011 [82]	A multicentre randomized double-blind placebo-controlled trial with the interleukin-1 receptor antagonist Anakinra in patients with systemic-onset juvenile idiopathic arthritis (ANAJIS trial).	Multicentre, randomized, double-blind, placebo-controlled trial	Pediatric (*n* = 24)	ANA (2 mg/kg s.c. daily, maximum 100 mg)
Ruperto et al. (trial 1), 2012 [83]	Two randomized trials of Canakinumab in systemic juvenile idiopathic arthritis.	2 phase III trials	Pediatric (*n* = 84 + 177)	CANA (s.c., 4 mg/kg per month (maximum dose, 300 mg))
Ruperto et al. (trial 2), 2012 [84]	A phase II, multicenter, open-label study evaluating dosing and preliminary safety and efficacy of Canakinumab in systemic juvenile idiopathic arthritis with active systemic features.	Phase II, multicenter, open-label, dosage-escalation study	Pediatric (*n* = 23)	CANA (single s.c. dose of 0.5 to 9 mg/kg)
Ruperto et al., 2018 [85]	Canakinumab in patients with systemic juvenile idiopathic arthritis and active systemic features: Results from the 5-year long-term extension of the phase III pivotal trials.	5-year long-term extension of the phase III pivotal trials.	Pediatric (*n* = 177; 144 in the long-term extension phase)	CANA (4 mg/kg s.c. every 4 weeks (maximum dose 300 mg); in the long-term extension, tapered to 2 mg/kg every 4 weeks in patients who were glucocorticoid free as per physicians’ judgement)

ANA: Anakinra; CANA: Canakinumab; IQR: interquartile range; n.a.: not applicable; s.c.: subcutaneous.

## References

[1] Teague M. (2017). Pediatric rheumatologic diseases: A review for primary care NPs. Nurse Pract..

[2] Rigante D. (2017). A systematic approach to autoinflammatory syndromes: A spelling booklet for the beginner. Expert Rev. Clin. Immunol..

[3] Cantarini L., Vitale A., Lucherini O.M., De Clemente C., Caso F., Costa L., Emmi G., Silvestri E., Magnotti F., Maggio M.C. (2015). The labyrinth of autoinflammatory disorders: A snapshot on the activity of a third-level center in Italy. Clin. Rheumatol..

[4] Rigante D., Lopalco G., Vitale A., Lucherini O.M., Caso F., De Clemente C., Molinaro F., Messina M., Costa L., Atteno M. (2014). Untangling the web of systemic autoinflammatory diseases. Mediat. Inflamm..

[5] Cattalini M., Soliani M., Lopalco G., Rigante D., Cantarini L. (2016). Systemic and organ involvement in monogenic autoinflammatory disorders: A global review filtered through internists’ lens. Intern. Emerg. Med..

[6] Obici L., Merlini G. (2012). Amyloidosis in autoinflammatory syndromes. Autoimmun. Rev..

[7] Cimaz R. (2016). Systemic-onset juvenile idiopathic arthritis. Autoimmun. Rev..

[8] Lee J.J.Y., Schneider R. (2018). Systemic juvenile idiopathic arthritis. Pediatr. Clin. N. Am..

[9] Barut K., Adrovic A., Şahin S., Kasapçopu Ö. (2017). Juvenile idiopathic arthritis. Balk. Med. J..

[10] Patel R.M., Shulman S.T. (2015). Kawasaki disease: A comprehensive review of treatment options. J. Clin. Pharm..

[11] Hersh A., von Scheven E., Yelin E. (2011). Adult outcomes of childhood-onset rheumatic diseases. Nat. Rev. Rheumatol..

[12] Weber A., Wasiliew P., Kracht M. (2010). Interleukin-1 (IL-1) pathway. Sci. Signal..

[13] Cantarini L., Lopalco G., Cattalini M., Vitale A., Galeazzi M., Rigante D. (2015). Interleukin-1: Ariadne’s Thread in autoinflammatory and autoimmune disorders. Isr. Med. Assoc. J..

[14] O’Neill L.A.J. (2008). The interleukin-1 receptor/Toll-like receptor superfamily: 10 years of progress. Immunol. Rev..

[15] Beesu M., Caruso G., Salyer A.C.D., Shukla N.M., Khetani K.K., Smith L.J., Fox L.M., Tanji H., Ohto U., Shimizu T. (2016). Identification of a human Toll-Like Receptor (TLR) 8-specific agonist and a functional pan-TLR inhibitor in 2-aminoimidazoles. J. Med. Chem..

[16] Federici S., Martini A., Gattorno M. (2013). The Central Role of Anti-IL-1 Blockade in the Treatment of Monogenic and Multi-Factorial Autoinflammatory Diseases. Front. Immunol..

[17] Dinarello C.A. (2018). Overview of the IL-1 family in innate inflammation and acquired immunity. Immunol. Rev..

[18] Masters S.L., Simon A., Aksentijevich I., Kastner D.L. (2009). Horror autoinflammaticus: The molecular pathophysiology of autoinflammatory disease (*). Annu. Rev. Immunol..

[19] Toplak N., Blazina Š., Avčin T. (2018). The role of IL-1 inhibition in systemic juvenile idiopathic arthritis: Current status and future perspectives. Drug Des. Dev. Ther..

[20] Bettiol A., Silvestri E., Di Scala G., Amedei A., Becatti M., Fiorillo C., Lopalco G., Salvarani C., Cantarini L., Soriano A. (2019). The right place of interleukin-1 inhibitors in the treatment of Behçet’s syndrome: A systematic review. Rheumatol. Int..

[21] European Medicines Agency Summary of Product Characteristics—Kineret. https://www.ema.europa.eu/documents/product-information/kineret-epar-product-information_en.pdf.

[22] Urien S., Bardin C., Bader-Meunier B., Mouy R., Compeyrot-Lacassagne S., Foissac F., Florkin B., Wouters C., Neven B., Treluyer J.-M. (2013). Anakinra pharmacokinetics in children and adolescents with systemic-onset juvenile idiopathic arthritis and autoinflammatory syndromes. BMC Pharmacol. Toxicol..

[23] European Medicines Agency Summary of Product Characteristics—Ilaris. https://www.ema.europa.eu/en/medicines/human/EPAR/ilaris.

[24] Sun H., Van L.M., Floch D., Jiang X., Klein U.R., Abrams K., Sunkara G. (2016). Pharmacokinetics and pharmacodynamics of canakinumab in patients with systemic juvenile idiopathic arthritis. J. Clin. Pharmacol..

[25] Cantarini L., Lucherini O.M., Frediani B., Brizi M.G., Bartolomei B., Cimaz R., Galeazzi M., Rigante D. (2011). Bridging the gap between the clinician and the patient with cryopyrin-associated periodic syndromes. Int. J. Immunopathol. Pharmacol..

[26] Lachmann H.J., Kone-Paut I., Kuemmerle-Deschner J.B., Leslie K.S., Hachulla E., Quartier P., Gitton X., Widmer A., Patel N., Hawkins P.N. (2009). Use of canakinumab in the cryopyrin-associated periodic syndrome. N. Engl. J. Med..

[27] Kuemmerle-Deschner J.B., Ramos E., Blank N., Roesler J., Felix S.D., Jung T., Stricker K., Chakraborty A., Tannenbaum S., Wright A.M. (2011). Canakinumab (ACZ885, a fully human IgG1 anti-IL-1β mAb) induces sustained remission in pediatric patients with cryopyrin-associated periodic syndrome (CAPS). Arthritis Res. Ther..

[28] Kuemmerle-Deschner J.B., Hachulla E., Cartwright R., Hawkins P.N., Tran T.A., Bader-Meunier B., Hoyer J., Gattorno M., Gul A., Smith J. (2011). Two-year results from an open-label, multicentre, phase III study evaluating the safety and efficacy of canakinumab in patients with cryopyrin-associated periodic syndrome across different severity phenotypes. Ann. Rheum. Dis..

[29] Koné-Paut I., Lachmann H.J., Kuemmerle-Deschner J.B., Hachulla E., Leslie K.S., Mouy R., Ferreira A., Lheritier K., Patel N., Preiss R. (2011). Sustained remission of symptoms and improved health-related quality of life in patients with cryopyrin-associated periodic syndrome treated with canakinumab: Results of a double-blind placebo-controlled randomized withdrawal study. Arthritis Res. Ther..

[30] Imagawa T., Nishikomori R., Takada H., Takeshita S., Patel N., Kim D., Lheritier K., Heike T., Hara T., Yokota S. (2013). Safety and efficacy of canakinumab in Japanese patients with phenotypes of cryopyrin-associated periodic syndrome as established in the first open-label, phase-3 pivotal study (24-week results). Clin. Exp. Rheumatol..

[31] Yokota S., Imagawa T., Nishikomori R., Takada H., Abrams K., Lheritier K., Heike T., Hara T. (2016). Long-term safety and efficacy of canakinumab in cryopyrin-associated periodic syndrome: Results from an open-label, phase III pivotal study in Japanese patients. Clin. Exp. Rheumatol..

[32] Kullenberg T., Löfqvist M., Leinonen M., Goldbach-Mansky R., Olivecrona H. (2016). Long-term safety profile of anakinra in patients with severe cryopyrin-associated periodic syndromes. Rheumatology.

[33] Goldbach-Mansky R., Dailey N.J., Canna S.W., Gelabert A., Jones J., Rubin B.I., Kim H.J., Brewer C., Zalewski C., Wiggs E. (2006). Neonatal-onset multisystem inflammatory disease responsive to interleukin-1beta inhibition. N. Engl. J. Med..

[34] Sibley C.H., Chioato A., Felix S., Colin L., Chakraborty A., Plass N., Rodriguez-Smith J., Brewer C., King K., Zalewski C. (2015). A 24-month open-label study of canakinumab in neonatal-onset multisystem inflammatory disease. Ann. Rheum. Dis..

[35] Wikén M., Hallén B., Kullenberg T., Koskinen L.O. (2018). Development and effect of antibodies to anakinra during treatment of severe CAPS: Sub-analysis of a long-term safety and efficacy study. Clin. Rheumatol..

[36] De Benedetti F., Gattorno M., Anton J., Ben-Chetrit E., Frenkel J., Hoffman H.M., Koné-Paut I., Lachmann H.J., Ozen S., Simon A. (2018). Canakinumab for the Treatment of Autoinflammatory Recurrent Fever Syndromes. N. Engl. J. Med..

[37] Gattorno M., Pelagatti M.A., Meini A., Obici L., Barcellona R., Federici S., Buoncompagni A., Plebani A., Merlini G., Martini A. (2008). Persistent efficacy of anakinra in patients with tumor necrosis factor receptor-associated periodic syndrome. Arthritis Rheum..

[38] Gattorno M., Obici L., Cattalini M., Tormey V., Abrams K., Davis N., Speziale A., Bhansali S.G., Martini A., Lachmann H.J. (2017). Canakinumab treatment for patients with active recurrent or chronic TNF receptor-associated periodic syndrome (TRAPS): An open-label, phase II study. Ann. Rheum. Dis..

[39] Torene R., Nirmala N., Obici L., Cattalini M., Tormey V., Caorsi R., Starck-Schwertz S., Letzkus M., Hartmann N., Abrams K. (2017). Canakinumab reverses overexpression of inflammatory response genes in tumour necrosis factor receptor-associated periodic syndrome. Ann. Rheum. Dis..

[40] Brik R., Butbul-Aviel Y., Lubin S., Ben Dayan E., Rachmilewitz-Minei T., Tseng L., Hashkes P.J. (2014). Canakinumab for the treatment of children with colchicine-resistant familial Mediterranean fever: A 6-month open-label, single-arm pilot study. Arthritis Rheumatol..

[41] Gül A., Ozdogan H., Erer B., Ugurlu S., Kasapcopur O., Davis N., Sevgi S. (2015). Efficacy and safety of canakinumab in adolescents and adults with colchicine-resistant familial Mediterranean fever. Arthritis Res. Ther..

[42] Arostegui J.I., Anton J., Calvo I., Robles A., Iglesias E., López-Montesinos B., Banchereau R., Hong S., Joubert Y., Junge G. (2017). Open-label phase II Study to assess the efficacy and safety of canakinumab treatment in active hyperimmunoglobulinemia D with periodic fever syndrome. Arthritis Rheumatol..

[43] Caroli F., Pontillo A., D’Osualdo A., Travan L., Ceccherini I., Crovella S., Alessio M., Stabile A., Gattorno M., Tommasini A. (2007). Clinical and genetic characterization of Italian patients affected by CINCA syndrome. Rheumatology.

[44] Neven B., Marvillet I., Terrada C., Ferster A., Boddaert N., Couloignier V., Pinto G., Pagnier A., Bodemer C., Bodaghi B. (2010). Long-term efficacy of the interleukin-1 receptor antagonist anakinra in ten patients with neonatal-onset multisystem inflammatory disease/chronic infantile neurologic, cutaneous, articular syndrome. Arthritis Rheum..

[45] Sibley C.H., Plass N., Snow J., Wiggs E.A., Brewer C.C., King K.A., Zalewski C., Kim H.J., Bishop R., Hill S. (2012). Sustained response and prevention of damage progression in patients with neonatal-onset multisystem inflammatory disease treated with anakinra: A cohort study to determine three- and five-year outcomes. Arthritis Rheum..

[46] Tran T. (2017). Muckle–Wells syndrome: Clinical perspectives. Open Access Rheumatol. Res. Rev..

[47] Kuemmerle-Deschner J.B., Tyrrell P.N., Koetter I., Wittkowski H., Bialkowski A., Tzaribachev N., Lohse P., Koitchev A., Deuter C., Foell D. (2011). Efficacy and safety of anakinra therapy in pediatric and adult patients with the autoinflammatory Muckle-Wells syndrome. Arthritis Rheum..

[48] Kuemmerle-Deschner J.B., Wittkowski H., Tyrrell P.N., Koetter I., Lohse P., Ummenhofer K., Reess F., Hansmann S., Koitschev A., Deuter C. (2013). Treatment of Muckle-Wells syndrome: Analysis of two IL-1-blocking regimens. Arthritis Res. Ther..

[49] Dalgic B., Egritas O., Sari S., Cuisset L. (2007). A variant Muckle-Wells syndrome with a novel mutation in CIAS1 gene responding to anakinra. Pediatr. Nephrol..

[50] Maksimovic L., Stirnemann J., Caux F., Ravet N., Rouaghe S., Cuisset L., Letellier E., Grateau G., Morin A.-S., Fain O. (2008). New *CIAS1* mutation and anakinra efficacy in overlapping of Muckle-Wells and familial cold autoinflammatory syndromes. Rheumatology.

[51] Marchica C., Zawawi F., Basodan D., Scuccimarri R., Daniel S.J. (2018). Resolution of unilateral sensorineural hearing loss in a pediatric patient with a severe phenotype of Muckle-Wells syndrome treated with Anakinra: A case report and review of the literature. J. Otolaryngol. Head Neck Surg..

[52] Stew B.T., Fishpool S.J.C., Owens D., Quine S. (2013). Muckle-Wells syndrome: A treatable cause of congenital sensorineural hearing loss. B-Ent.

[53] Yamazaki T., Masumoto J., Agematsu K., Sawai N., Kobayashi S., Shigemura T., Yasui K., Koike K. (2008). Anakinra improves sensory deafness in a Japanese patient with Muckle-Wells syndrome, possibly by inhibiting the cryopyrin inflammasome. Arthritis Rheum..

[54] Rigante D., Lopalco G., Vitale A., Lucherini O.M., De Clemente C., Caso F., Emmi G., Costa L., Silvestri E., Andreozzi L. (2014). Key facts and hot spots on tumor necrosis factor receptor-associated periodic syndrome. Clin. Rheumatol..

[55] Magnotti F., Vitale A., Rigante D., Lucherini O.M., Cimaz R., Muscari I., Granados Afonso de Faria A., Frediani B., Galeazzi M., Cantarini L. (2013). The most recent advances in pathophysiology and management of tumour necrosis factor receptor-associated periodic syndrome (TRAPS): Personal experience and literature review. Clin. Exp. Rheumatol..

[56] Lopalco G., Rigante D., Vitale A., Frediani B., Iannone F., Cantarini L. (2015). Tumor necrosis factor receptor-associated periodic syndrome managed with the couple canakinumab-alendronate. Clin. Rheumatol..

[57] Cantarini L., Lopalco G., Vitale A., Caso F., Lapadula G., Iannone F., Galeazzi M., Rigante D. (2015). Delights and let-downs in the management of tumor necrosis factor receptor-associated periodic syndrome: The canakinumab experience in a patient with a high-penetrance T50M *TNFRSF1A* variant. Int. J. Rheum. Dis..

[58] Rigante D., Lopalco G., Tarantino G., Compagnone A., Fastiggi M., Cantarini L. (2015). Non-canonical manifestations of familial Mediterranean fever: A changing paradigm. Clin. Rheumatol..

[59] Alghamdi M. (2017). Familial Mediterranean fever, review of the literature. Clin. Rheumatol..

[60] Gülez N., Makay B., Sözeri B. (2018). Long-Term Effectıveness and safety of canakınumab ın pedıatrıc famılıal Medıterranean fever patıents. Mod. Rheumatol..

[61] Laskari K., Boura P., Dalekos G.N., Garyfallos A., Karokis D., Pikazis D., Settas L., Skarantavos G., Tsitsami E., Sfikakis P.P. (2017). Long-term beneficial effect of canakinumab in colchicine-resistant familial Mediterranean fever. J. Rheumatol..

[62] Başaran Ö., Uncu N., Çelikel B.A., Taktak A., Gür G., Cakar N. (2015). Interleukin-1 targeting treatment in familial Mediterranean fever: An experience of pediatric patients. Mod. Rheumatol..

[63] Cetin P., Sari I., Sozeri B., Cam O., Birlik M., Akkoc N., Onen F., Akar S. (2015). Efficacy of Interleukin-1 Targeting Treatments in Patients with Familial Mediterranean Fever. Inflammation.

[64] Özçakar Z.B., Özdel S., Yılmaz S., Kurt-Şükür E.D., Ekim M., Yalçınkaya F. (2016). Anti-IL-1 treatment in familial Mediterranean fever and related amyloidosis. Clin. Rheumatol..

[65] Houten S.M., Kuis W., Duran M., de Koning T.J., van Royen-Kerkhof A., Romeijn G.J., Frenkel J., Dorland L., de Barse M.M.J., Huijbers W.A.R. (1999). Mutations in *MVK*, encoding mevalonate kinase, cause hyperimmunoglobulinaemia D and periodic fever syndrome. Nat. Genet..

[66] Ter Haar N.M., Jeyaratnam J., Lachmann H.J., Simon A., Brogan P.A., Doglio M., Cattalini M., Anton J., Modesto C., Quartier P. (2016). The phenotype and genotype of mevalonate kinase deficiency: A series of 114 cases from the Eurofever registry. Arthritis Rheumatol..

[67] Bodar E.J., Kuijk L.M., Drenth J.P.H., van der Meer J.W.M., Simon A., Frenkel J. (2011). On-demand anakinra treatment is effective in mevalonate kinase deficiency. Ann. Rheum. Dis..

[68] van der Hilst J.C.H., Bodar E.J., Barron K.S., Frenkel J., Drenth J.P.H., van der Meer J.W.M., Simon A. (2008). International HIDS Study Group Long-term follow-up, clinical features, and quality of life in a series of 103 patients with hyperimmunoglobulinemia D syndrome. Medicine.

[69] Galeotti C., Meinzer U., Quartier P., Rossi-Semerano L., Bader-Meunier B., Pillet P., Koné-Paut I. (2012). Efficacy of interleukin-1-targeting drugs in mevalonate kinase deficiency. Rheumatology.

[70] Tanaka T., Yoshioka K., Nishikomori R., Sakai H., Abe J., Yamashita Y., Hiramoto R., Morimoto A., Ishii E., Arakawa H. (2018). National survey of Japanese patients with mevalonate kinase deficiency reveals distinctive genetic and clinical characteristics. Mod. Rheumatol..

[71] Ozen S., Kuemmerle-Deschner J.B., Cimaz R., Livneh A., Quartier P., Kone-Paut I., Zeft A., Spalding S., Gul A., Hentgen V. (2017). International retrospective chart review of treatment patterns in severe familial Mediterranean fever, tumor necrosis factor receptor-associated periodic syndrome, and mevalonate kinase deficiency/hyperimmunoglobulinemia D syndrome. Arthritis Care Res..

[72] Ter Haar N., Lachmann H., Özen S., Woo P., Uziel Y., Modesto C., Koné-Paut I., Cantarini L., Insalaco A., Neven B. (2013). Treatment of autoinflammatory diseases: Results from the Eurofever Registry and a literature review. Ann. Rheum. Dis..

[73] Vitale A., Insalaco A., Sfriso P., Lopalco G., Emmi G., Cattalini M., Manna R., Cimaz R., Priori R., Talarico R. (2016). A snapshot on the on-label and off-label use of the interleukin-1 inhibitors in Italy among rheumatologists and pediatric rheumatologists: A nationwide multi-center retrospective observational study. Front. Pharmacol..

[74] Rossi-Semerano L., Fautrel B., Wendling D., Hachulla E., Galeotti C., Semerano L., Touitou I., Koné-Paut I. (2015). Tolerance and efficacy of off-label anti-interleukin-1 treatments in France: A nationwide survey. Orphanet J. Rare Dis..

[75] Topaloglu R., Batu E.D., Orhan D., Ozen S., Besbas N. (2016). Anti-interleukin 1 treatment in secondary amyloidosis associated with autoinflammatory diseases. Pediatr. Nephrol..

[76] Gattorno M., Piccini A., Lasigliè D., Tassi S., Brisca G., Carta S., Delfino L., Ferlito F., Pelagatti M.A., Caroli F. (2008). The pattern of response to anti-interleukin-1 treatment distinguishes two subsets of patients with systemic-onset juvenile idiopathic arthritis. Arthritis Rheum..

[77] Brachat A.H., Grom A.A., Wulffraat N., Brunner H.I., Quartier P., Brik R., McCann L., Ozdogan H., Rutkowska-Sak L., Schneider R. (2017). Early changes in gene expression and inflammatory proteins in systemic juvenile idiopathic arthritis patients on canakinumab therapy. Arthritis Res. Ther..

[78] Feist E., Quartier P., Fautrel B., Schneider R., Sfriso P., Efthimiou P., Cantarini L., Lheritier K., Leon K., Karyekar C.S. (2018). Efficacy and safety of canakinumab in patients with Still’s disease: Exposure-response analysis of pooled systemic juvenile idiopathic arthritis data by age groups. Clin. Exp. Rheumatol..

[79] Grom A.A., Ilowite N.T., Pascual V., Brunner H.I., Martini A., Lovell D., Ruperto N., Abrams K., Leon K., Lheritier K. (2016). Rate and clinical presentation of macrophage activation syndrome in patients with systemic juvenile idiopathic arthritis treated with canakinumab. Arthritis Rheumatol..

[80] Ilowite N., Porras O., Reiff A., Rudge S., Punaro M., Martin A., Allen R., Harville T., Sun Y.-N., Bevirt T. (2009). Anakinra in the treatment of polyarticular-course juvenile rheumatoid arthritis: Safety and preliminary efficacy results of a randomized multicenter study. Clin. Rheumatol..

[81] Kimura Y., Grevich S., Beukelman T., Morgan E., Nigrovic P.A., Mieszkalski K., Graham T.B., Ibarra M., Ilowite N., Klein-Gitelman M. (2017). Pilot study comparing the Childhood Arthritis & Rheumatology Research Alliance (CARRA) systemic Juvenile Idiopathic Arthritis consensus treatment plans. Pediatr. Rheumatol. Online J..

[82] Quartier P., Allantaz F., Cimaz R., Pillet P., Messiaen C., Bardin C., Bossuyt X., Boutten A., Bienvenu J., Duquesne A. (2011). A multicentre, randomised, double-blind, placebo-controlled trial with the interleukin-1 receptor antagonist anakinra in patients with systemic-onset juvenile idiopathic arthritis (ANAJIS trial). Ann. Rheum. Dis..

[83] Ruperto N., Brunner H.I., Quartier P., Constantin T., Wulffraat N., Horneff G., Brik R., McCann L., Kasapcopur O., Rutkowska-Sak L. (2012). Two randomized trials of canakinumab in systemic juvenile idiopathic arthritis. N. Engl. J. Med..

[84] Ruperto N., Quartier P., Wulffraat N., Woo P., Ravelli A., Mouy R., Bader-Meunier B., Vastert S.J., Noseda E., D’Ambrosio D. (2012). A phase II, multicenter, open-label study evaluating dosing and preliminary safety and efficacy of canakinumab in systemic juvenile idiopathic arthritis with active systemic features. Arthritis Rheum..

[85] Ruperto N., Brunner H.I., Quartier P., Constantin T., Wulffraat N.M., Horneff G., Kasapcopur O., Schneider R., Anton J., Barash J. (2018). Canakinumab in patients with systemic juvenile idiopathic arthritis and active systemic features: Results from the 5-year long-term extension of the phase III pivotal trials. Ann. Rheum. Dis..

[86] Hedrich C.M., Bruck N., Fiebig B., Gahr M. (2012). Anakinra: A safe and effective first-line treatment in systemic onset juvenile idiopathic arthritis (SoJIA). Rheumatol. Int..

[87] Horneff G., Schulz A.C., Klotsche J., Hospach A., Minden K., Foeldvari I., Trauzeddel R., Ganser G., Weller-Heinemann F., Haas J.P. (2017). Experience with etanercept, tocilizumab and interleukin-1 inhibitors in systemic onset juvenile idiopathic arthritis patients from the BIKER registry. Arthritis Res. Ther..

[88] Kearsley-Fleet L., Beresford M.W., Davies R., De Cock D., Baildam E., Foster H.E., Southwood T.R., Thomson W., Hyrich K.L. (2019). Short-term outcomes in patients with systemic juvenile idiopathic arthritis treated with either tocilizumab or anakinra. Rheumatology.

[89] Lequerré T., Quartier P., Rosellini D., Alaoui F., De Bandt M., Mejjad O., Koné-Paut I., Michel M., Dernis E., Khellaf M. (2008). Interleukin-1 receptor antagonist (anakinra) treatment in patients with systemic-onset juvenile idiopathic arthritis or adult onset Still disease: Preliminary experience in France. Ann. Rheum. Dis..

[90] Nigrovic P.A., Mannion M., Prince F.H.M., Zeft A., Rabinovich C.E., van Rossum M.A.J., Cortis E., Pardeo M., Miettunen P.M., Janow G. (2011). Anakinra as first-line disease-modifying therapy in systemic juvenile idiopathic arthritis: Report of forty-six patients from an international multicenter series. Arthritis Rheum..

[91] Pardeo M., Pires Marafon D., Insalaco A., Bracaglia C., Nicolai R., Messia V., De Benedetti F. (2015). Anakinra in systemic juvenile idiopathic arthritis: A single-center experience. J. Rheumatol..

[92] Romano M., Pontikaki I., Gattinara M., Ardoino I., Donati C., Boracchi P., Meroni P.L., Gerloni V. (2014). Drug survival and reasons for discontinuation of the first course of biological therapy in 301 juvenile idiopathic arthritis patients. Reumatismo.

[93] Vastert S.J., de Jager W., Noordman B.J., Holzinger D., Kuis W., Prakken B.J., Wulffraat N.M. (2014). Effectiveness of first-line treatment with recombinant interleukin-1 receptor antagonist in steroid-naive patients with new-onset systemic juvenile idiopathic arthritis: Results of a prospective cohort study. Arthritis Rheumatol..

[94] Marrani E., Burns J.C., Cimaz R. (2018). How should we classify Kawasaki disease?. Front. Immunol..

[95] Principi N., Rigante D., Esposito S. (2013). The role of infection in Kawasaki syndrome. J. Infect..

[96] De Rosa G., Pardeo M., Rigante D. (2007). Current recommendations for the pharmacologic therapy in Kawasaki syndrome and management of its cardiovascular complications. Eur. Rev. Med. Pharmacol. Sci..

[97] Rigante D., Valentini P., Rizzo D., Leo A., De Rosa G., Onesimo R., De Nisco A., Angelone D.F., Compagnone A., Delogu A.B. (2010). Responsiveness to intravenous immunoglobulins and occurrence of coronary artery abnormalities in a single-center cohort of Italian patients with Kawasaki syndrome. Rheumatol. Int..

[98] Rigante D., Andreozzi L., Fastiggi M., Bracci B., Natale M.F., Esposito S. (2016). Critical overview of the risk scoring systems to predict non-responsiveness to intravenous immunoglobulin in Kawasaki syndrome. Int. J. Mol. Sci..

[99] Agarwal S., Agrawal D.K. (2017). Kawasaki disease: Etiopathogenesis and novel treatment strategies. Expert Rev. Clin. Immunol..

[100] Blonz G., Lacroix S., Benbrik N., Warin-Fresse K., Masseau A., Trewick D., Hamidou M., Stephan J.-L., Néel A. (2018). Severe late-onset Kawasaki disease successfully treated with anakinra. J. Clin. Rheumatol..

[101] Cohen S., Tacke C.E., Straver B., Meijer N., Kuipers I.M., Kuijpers T.W. (2012). A child with severe relapsing Kawasaki disease rescued by IL-1 receptor blockade and extracorporeal membrane oxygenation. Ann. Rheum. Dis..

[102] Guillaume M.-P., Reumaux H., Dubos F. (2018). Usefulness and safety of anakinra in refractory Kawasaki disease complicated by coronary artery aneurysm. Cardiol. Young.

[103] Sánchez-Manubens J., Gelman A., Franch N., Teodoro S., Palacios J.R., Rudi N., Rivera J., Antón J. (2017). A child with resistant Kawasaki disease successfully treated with anakinra: A case report. BMC Pediatr..

[104] Shafferman A., Birmingham J.D., Cron R.Q. (2014). High dose Anakinra for treatment of severe neonatal Kawasaki disease: A case report. Pediatr. Rheumatol. Online J..

[105] Koné-Paut I., Cimaz R., Herberg J., Bates O., Carbasse A., Saulnier J.P., Maggio M.C., Anton J., Piram M. (2018). The use of interleukin 1 receptor antagonist (anakinra) in Kawasaki disease: A retrospective cases series. Autoimmun. Rev..

[106] Kaiser C., Knight A., Nordström D., Pettersson T., Fransson J., Florin-Robertsson E., Pilström B. (2012). Injection-site reactions upon Kineret (anakinra) administration: Experiences and explanations. Rheumatol. Int..

[107] Sota J., Vitale A., Insalaco A., Sfriso P., Lopalco G., Emmi G., Cattalini M., Manna R., Cimaz R., Priori R. (2018). Safety profile of the interleukin-1 inhibitors anakinra and canakinumab in real-life clinical practice: A nationwide multicenter retrospective observational study. Clin. Rheumatol..

